# Selective attention to stimulus representations in perception and memory: commonalities and differences

**DOI:** 10.1007/s00426-020-01469-z

**Published:** 2021-01-24

**Authors:** Jasmin M. Kizilirmak, Sarah Glim, Margarita Darna, Patrick H. Khader

**Affiliations:** 1 Cognitive Neuroscience Lab, Institute of Psychology, University of Hildesheim, Universitätsplatz 1, 31141 Hildesheim, Germany; 2 German Center for Neurodegenerative Diseases, Von-Siebold-Star. 3a, 37075 Göttingen, Germany; 3 Institute for Psychology, University of Kassel, Holländische Str. 36-38, 34127 Kassel, Germany; 4 Georg-August-Universität Göttingen, Wilhelmsplatz 1, 37073 Göttingen, Germany; 5 Psychology School, Fresenius University of Applied Sciences, Frankfurt a. M., Germany

## Abstract

It has been proposed that the deployment of selective attention to perceptual and memory representations might be governed by similar cognitive processes and neural resources. However, evidence for this simple and appealing proposal remains inconclusive, which might be due to a considerable divergence in tasks and cognitive demands when comparing attentional selection in memory versus perception. To examine whether selection in both domains share common attentional processes and only differ in the stimuli they act upon (external vs. internal), we compared behavioral costs or benefits between selection domains. In both domains, participants had to attend a target stimulus from a set of simultaneously presented stimuli or simultaneously active memory representations, respectively, with set, target, or both, being repeated or changed across trials. The results of two experiments delineated principal similarities and differences of selection processes in both domains: While positive priming from stimulus repetition was found in both selection domains, we found no consistent effects of negative priming when shifting the focus of attention to a previously to-be-ignored stimulus. However, priming in the perception task was mainly due to repetitions of the target feature (here: color), whereas for the memory task, repetition of the same set of stimulus representations was most important. We propose that the differences can be attributed to a reduced cognitive effort when the now relevant memory representation had already been pre-activated (even as a distractor) in the previous trial. Additionally, our experiments both underscore the importance of taking stimulus–response associations into account, which may be a hidden factor behind differences between domains. We conclude that any attempt of comparing internal versus external attentional selection has to consider inherent differences in selection dynamics across representational domains.

## Introduction

Attentional selection has been investigated in perception (selective attention; e.g., Driver, 2001; Moore & Zirnsak, 2017) and memory (selective retrieval; e.g., Buckner & Wheeler, 2001; Eichenbaum, 2017; Mecklinger, 2010). Moreover, there is evidence that one influences the other, such as disengaging the gaze to withdraw attention from the external world to facilitate its focus on internal memory representations (Glenberg, Schroeder, & Robertson, 1998), or that the novelty or familiarity of an item affects how long it captures attention (Parks & Hopfinger, 2008). However, it is unclear whether each domain has its own selection process or whether selection in both domains shares a common neurocognitive basis, only differing in the stimuli it acts upon (external sensory environment vs. internal memory space). It has been repeatedly observed that the deployment of attention to sensory input and to representations in long-term memory (LTM) is governed by similar neurocognitive processes (Cabeza, 2008; Cabeza et al., 2003). Specifically, lesions in the posterior parietal cortex, especially the inferior parietal lobe, are associated with attentional selection as well as LTM retrieval deficits (Berryhill, 2012; Finke, Myers, Bublak, & Sorg, 2013; Hower, Wixted, Berryhill, & Olson, 2014). The attention-to-memory (AtoM) theory attempts to explain this commonality by proposing the same brain regions to be involved in attentional selection in perception and in LTM, i.e., in external versus. internal representational space (Cabeza, Ciaramelli, Olson, & Moscovitch, 2008; Ciaramelli, Grady, & Moscovitch, 2008).

While numerous studies support the AtoM theory, there are also findings that challenge it. For example, in their review of the contribution of the posterior parietal cortex (PPC) to episodic memory, Sestieri, Shulman, and Corbetta (2017) argue against a complete anatomical and functional overlap between attention to memory and perception. Furthermore, research on the fronto-parietal network of attentional control suggests that different subcomponents of the network are involved in different attentional demands (Cole et al., 2013; Corbetta & Shulman, 2002; Wang et al., 2010; Zanto & Gazzaley, 2013). For example, Dixon et al. (2018) propose, based on an extensive meta-analysis, that at least two functionally and anatomically distinct sub-networks of the fronto-parietal network can be differentiated, one associated with more *internally* oriented attention, such as mentalizing and emotional processing, and the other one with more *externally* oriented attention, such as reading and sensory-motor tasks.

Note that virtually all of the attempts to compare selective attention in memory versus perception did this across different studies with different stimulus materials and tasks (Hutchinson, Uncapher, & Wagner 2009, 2014; Sestieri Shulman, & Corbetta, 2010). In fact, we know of only one study that tried to compare attention to perception and memory using the same stimulus material and a comparable task. Sestieri et al. (2010) employed short video clips for both a perceptual as well as a memory-search paradigm in functional magnetic resonance imaging (fMRI) study. Their results showed adjacent, but distinct regions to be activated by search processes in memory and perception. However, one needs to take into consideration that at least part of the discrepancies in activation patterns might have resulted from systematic differences in the applied experimental tasks, such as stimulus material and the demand on cognitive-control processes, rather than from differences in the underlying brain mechanisms.

## Developing a paradigm suited to directly compare attention to memory versus perception.

In the present study, we developed two variants of a behavioral paradigm to study the attentional control processes involved in selective stimulus processing in perception versus memory within subjects. The respective processes were probed by introducing different levels of interference across trials. Typical paradigms to assess this kind of interference to trigger attentional control employ trials during which several stimuli are presented simultaneously. Usually, one stimulus out of this stimulus set is task-relevant (target) while the others are distractors that have to be ignored (e.g., Egner & Hirsch, 2005; Stadler & Hogan, 1996; Tipper & Driver, 1988). To evaluate what kind of control processes were applied to the target representation and the distractors, the following trial-to-trial effects are compared: (1) repetition of the target (and/or distractor/s), (2) former distractor becoming the target (and/or former target becoming a distractor), and (3) all stimuli change. The third case is thought to be a neutral control condition, in which neither beneficial nor detrimental effects from the preceding trial should be observed. Typical findings are a response time (RT) benefit for repetitions of the target (positive priming) and prolonged RTs for switches between targets and distractors (negative priming; e.g., Tipper & Driver, 1988), especially when a previous distractor becomes a target in the immediately following trial. The most common interpretation of these effects is to assume attentional selection of targets and inhibition of distractors. This two-process account is typically referred to as the *inhibition account* (Tipper, 2001).

In memory, cognitive control during selective retrieval is often studied by having participants learn several associations with the same cue (usually category-exemplar associations), and then having them selectively retrieve some (retrieval practice: Rp^+^) but not others (Rp^−^). Normally, there is also a neutral control condition in which no association has to be selectively retrieved after initial learning (N). The manipulation is list-wise, not trial-wise as in selective perception. This paradigm is called retrieval-practice paradigm and was established by Anderson, Bjork, and Bjork (1994). When comparing the three conditions at the end of the procedure (1. learning phase of all cues and their associated items, 2. retrieval practice of Rp^+^ items via cued recall, 3. final test of all items via cued recall), the typical finding^1^ is the following order of retrieval success: Rp^+^  > N > Rp^−^. This pattern has long been interpreted as evidence for inhibition in memory and has been termed retrieval-induced forgetting (RIF) (Anderson et al. 1994; Ciranni & Shimamura, 1999). Evidence from paradigms testing memory for the retrieval competitors with alternative cues suggests that the representation of retrieval competitors itself had been weakened, which is taken as additional support for the inhibition account (Anderson & Spellman, 1995; Johnson & Anderson, 2004).

**Fig. 1:**
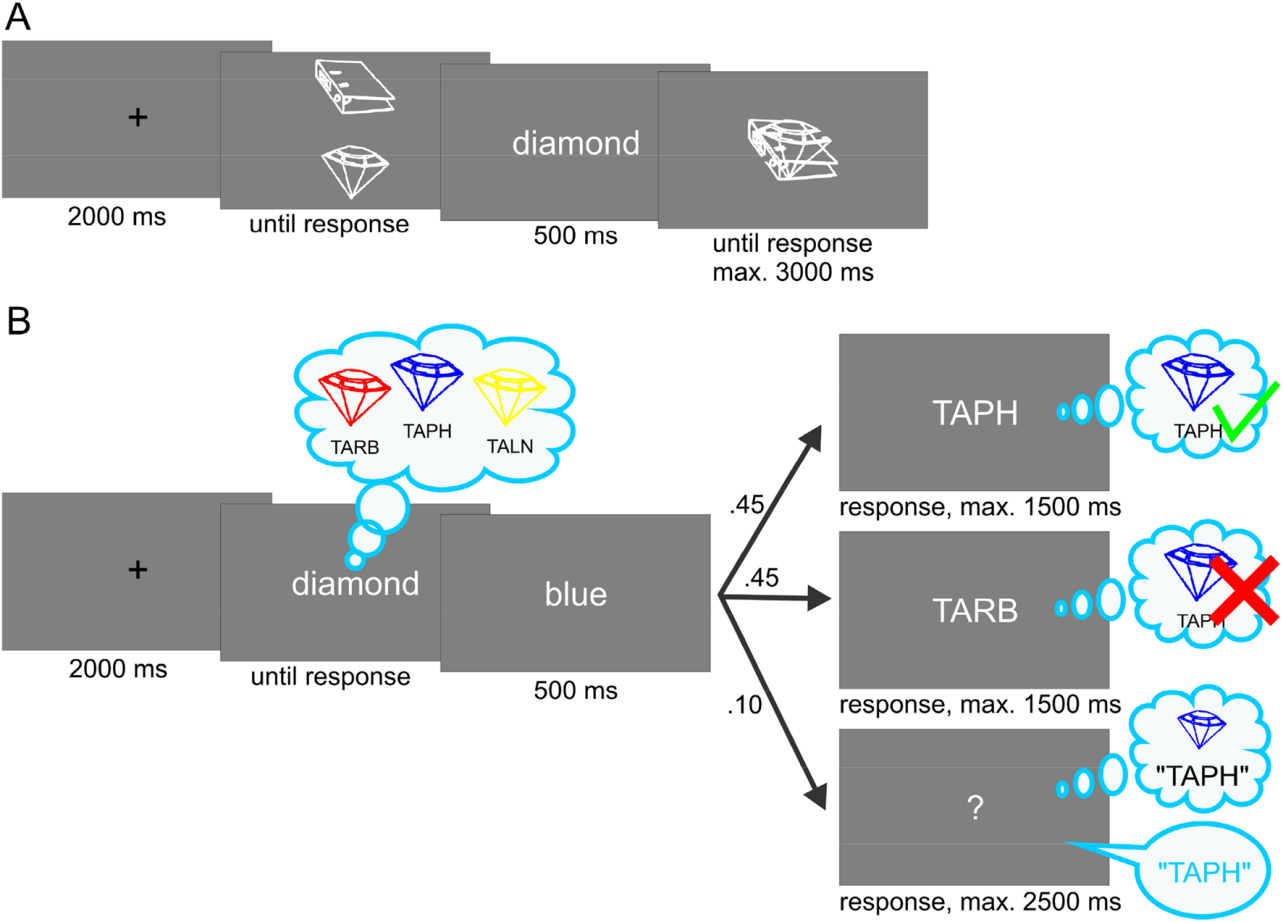
Exemplary trials of the selective visual perception task (**a**) and the selective long-term memory retrieval task (**b**). As can be seen by comparing both panels, the task procedure was parallelized as far as possible by (1) using the same line-drawings as stimuli, (2) always first activating a set of potentially task-relevant representations (external or internal, respectively), and then (3) cueing the target and thereby prompting selective attention

Based on these considerations of beneficial and detrimental effects across trials, we present a novel attentional-selection paradigm for the investigation of attention to percepts and memories based on positive and negative priming (e.g., Schrobsdorff et al., 2007; Stadler & Hogan, 1996) that carefully matched the demands on cognitive-control processes while keeping stimulus material and task requirements as comparable as possible. To this end, we conducted two experiments in which selection in perception and memory were matched to fulfill various criteria. In both experiments, we used line drawings of objects which could be grouped into categories on which selection had to be carried out. In the first experiment, the main focus was on parallelizing the timing of the processing steps as closely as possible, while sticking closely to classical experimental positive/negative priming and selective long-term memory retrieval designs. One problematic aspect was that while representations of the stimuli are processed online for perception, they first have to be activated in memory to perform any additional process on them. Hence, to ensure that only the selection was carried out during the critical display, in experiment 1, retrieval of all potentially relevant stimuli (targets and distractors) was carried out at the beginning of each trial, prior to the cue indicating the target (Fig. 1). In experiment 2, we focused more strongly on parallelizing the exact number and appearance of the stimuli for the perception and memory tasks (Fig. 5). Inter-trial effects were examined in both domains by systematically repeating or changing the underlying set of potentially response-relevant internal/external representations, as well as repetitions of the target and distractors switching their roles.

Based on the reported findings on positive and negative priming in visual attention tasks (Stadler & Hogan, 1996), we expected shorter RTs and/or reduced error rates (compared to a complete change of the target and distractor stimuli) when both the target and distractors were repeated as well as when the target was repeated while the distractor changed. In contrast, a repetition of the set of stimuli but with a swap of their roles (former target becomes distractor and vice versa) should be associated with increased RTs and/or error rates. In case of a strong correspondence between attentional selection in perception and memory, we would predict similar effects for the selective LTM retrieval task. In accordance with positive priming, we should find facilitative effects on RTs in the LTM task when the retrieval target was being repeated. In contrast to negative priming, which in the selective LTM retrieval task would correspond to detrimental effects of switches to a previously irrelevant association with the same cue as in trial *i*-1, we expected no negative consequences or even beneficial effects due to spreading activation. This prediction was based on our previous findings (Kizilirmak, Rösler, & Khader, 2014) and would be in line with the found differences between attention-related brain regions for perception versus LTM retrieval (e.g., Hutchinson et al., 2009).

## Experiment 1

Figure 1 illustrates the perceptual and memory selection tasks. As can be seen by comparing both panels, the task procedures were parallelized as far as possible: Both, the selective perception and selective LTM retrieval task (1) used the same line-drawings as stimuli, (2) always activated a set of potentially task-relevant representations (external or internal, respectively) first and then (3) cued the target, prompting selective attention (see Fig. 1). Step 2 was implemented to ensure that differences could not be attributed to the one additional step required in memory retrieval: the activation of the memory representations, that is, the *uploading* into a working-memory buffer for online processing.

The different conditions were based on different demands on selective attention from trial *i*−1 to *i*. For both tasks, we included a control condition, which should be free from effects of any control processes (enhancement of the target and/or inhibition of distractors) applied in the previous trial. To this end, the complete set of stimuli changed from trial *i*−1 to trial *i*. The other conditions varied systematically with respect to sets and/or target stimuli being repeated or changed, which are typical conditions under which positive and negative priming can be observed.

## Methods

### Participants

Twenty-five volunteers (18 female) gave informed consent to participate in the study. Participants were 20–39 years old (*Mdn* = 24 years, *SD* = 4.9 years). Twenty-three were right-handed and two were left-handed by self-report. They either participated for course credits or were paid 8 € per hour, had normal or corrected-to-normal vision, and were naïve regarding the hypotheses of the study. Two participants had to be excluded because they missed the criterion of 60% correct responses in any condition of the visual perception task. The final sample comprised 23 participants (17 female) between 20 and 39 years old (*Mdn* = 26 years, *SD* = 4.9 years; 21 right-handed).

### Apparatus and procedure

Participants were tested individually in a dimly lit room, positioned at approximately 57 cm from a standard 22" ASUS TFT monitor with a resolution of 1920 × 1080 pixels and a refresh rate of 60 Hz. The experiment was run on a Windows XP operating system using the Psychophysics Toolbox Version 2.54 (Brainard, 1997; Pelli, 1997) in Matlab 7.5.0 (R2007b; The MathWorks, Inc., Natick, MA). Manual responses were recorded with a standard USB computer keyboard and a standard USB computer mouse. Verbal responses were noted down by the experimenter during the session. The visual perception task was always run before the LTM task, because it was expected to produce fewer undesired carry-over effects, such as retrieving the verbal labels, which became associated to the different objects in the LTM task. Trials of each task were presented in randomized order.

### Selective perception task

#### Stimulus material

**Fig. 2:**
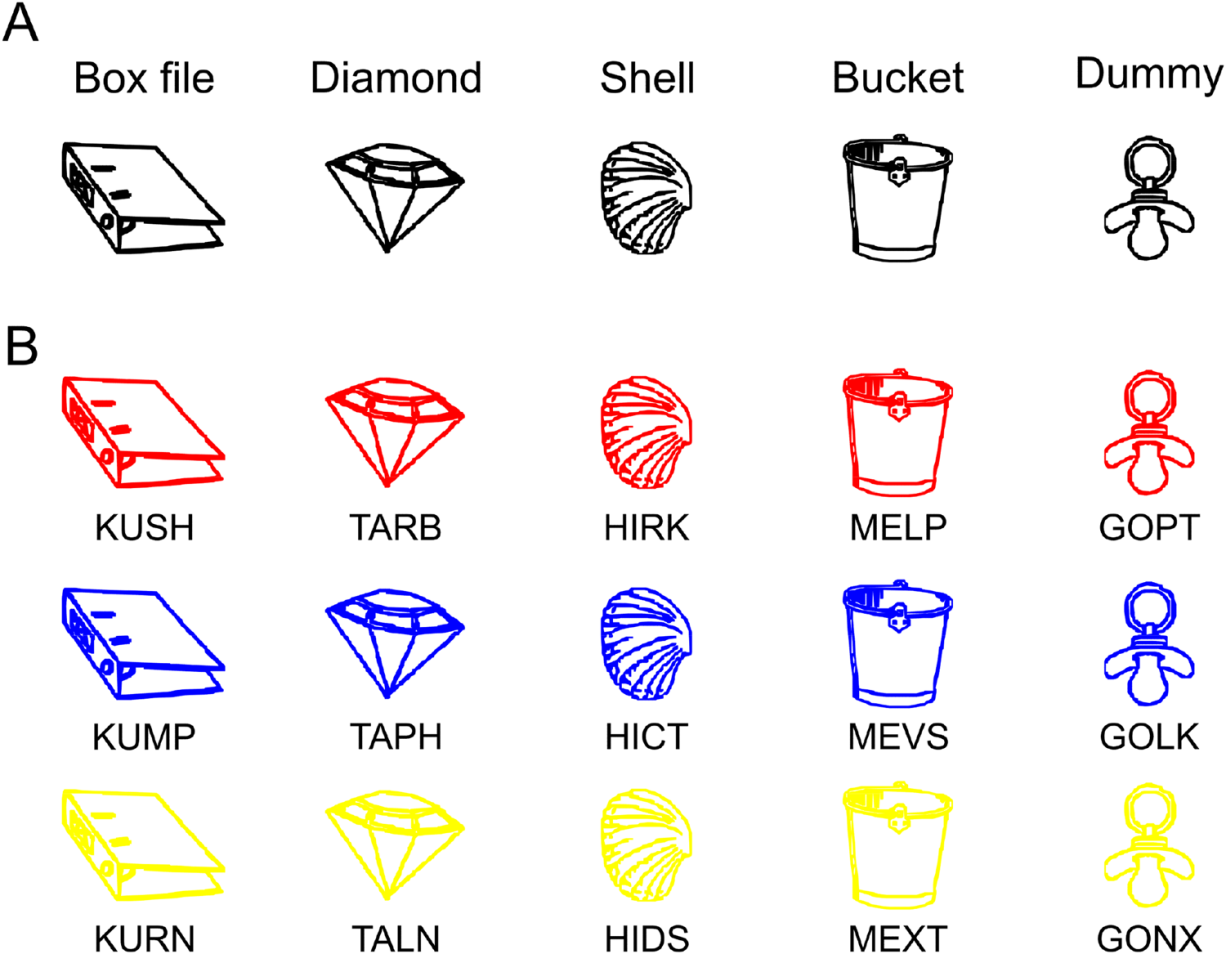
Stimuli of the visual selective attention (**a**) and selective retrieval task (**b**). The stimuli of the selective LTM task are presented along with their nonword names

The stimulus material used in the selective perception task consisted of five different line drawings of objects (see Fig. 2a). These drawings were slightly modified versions of pictures taken from the Bonin, Peereman, Malardier, Méot, and Chalard (2003) picture set. Pictures were selected based on comparable subjective visual complexity ratings as provided by Bonin and colleagues. The selected drawings had a mean visual complexity of 2.10 (*SD* = 0.06) on a scale from 1 (“very simple drawing”) to 5 (“very complex drawing”; see Bonin et al., 2003) and each drawing belonged to a different semantic category. The drawings were presented in white on a gray background (RGB code: 128-128-128) with a mean width of 3.70 cm (*SD* = 0.99 cm) and a mean height of 3.64 cm (*SD* = 0.21 cm). In the discrimination display (see "Procedure" of this task), the lines of each drawing were interrupted by a small gap with a height of 0.13 cm, located at a fixed position either on the drawing’s left or on its right side. This gap is what participants had to detect.

#### Procedure

The first phase was administered to familiarize the participants with the stimuli. Each drawing was presented centrally for 3000 ms with its English^2^ name appearing above the drawing in white. The order of stimuli was randomized for each participant. Then, the actual selective perception task started (see Fig. 1a). Each trial began with a centrally presented fixation cross for 2000 ms. Next, two line-drawings were presented, one above and the other one below the screen’s center. The allocation of drawings to positions was randomized. Participants were instructed to pay close attention to the drawings as they were both potentially relevant to the upcoming task and to press the space bar once they had taken a close look. As noted above, this step was implemented to increase the comparability to the selective LTM task in which participants were instructed to recall all drawings associated with the category cue plus their names (e.g., all diamonds and their names as depicted in Fig. 2b). After the presentation of the two drawings, the name of the target drawing appeared centrally for 500 ms. In the following discrimination display, both drawings were presented superimposed at the same location and each of them had a small gap either on its left or on its right side. Participants were instructed to press either the left or right button of a computer mouse, depending on which side the gap of the target drawing was. They were to do so as quickly and accurately as possible. The combination of gap locations (left/left, left/right, right/left, right/right) was chosen randomly for each trial.

The relative positions of the drawings with respect to each other were held constant to ensure that the gaps could unambiguously be attributed to the drawings, but their absolute position on the screen varied from trial to trial: The two drawings were randomly centered on one of ten possible coordinates that were arranged equidistantly on an imaginary circle with a radius of 0.51 cm around the center of the screen. This variation was introduced to prevent an anticipatory allocation of spatial attention to a specific location. Immediately after the response, but not later than 3000 ms after stimulus onset (timeout), the discrimination display was replaced by a fixation cross and the next trial started. A response timeout occurred in *M* = 1.76% of all trials, varying from 0 to 9.42% across participants.

Each block of the perception task consisted of 26 trials. The task started with a practice block that was not included in the analysis, followed by 20 experimental blocks (546 trials in total). During practice, incorrect responses were followed by the word “Error”, which was presented centrally for 1000 ms in RGB red (255-0-0). During the experiment proper, no feedback was provided. Participants started each block manually by pressing the space bar to allow for short breaks between blocks. Average task duration was 60 min.

#### Design

To examine trial-by-trial effects of attentional selection, the drawings varied systematically across trials, inducing different priming conditions. On each block’s first trial, both the target and the distractor were chosen randomly from the pool of line drawings. The remaining 25 trials per block were equally assigned to the following conditions (see also Table 1 for examples): In the control condition, the set (S) of stimuli (target and distractor) was changed from trial *i*−1 to *i*, resulting also in a target (T) change (S_ch/T_ch). There were two stimulus set *repetition* (S_rep) conditions, one in which target and distractor kept their roles (S_rep/T_rep; also considered as full positive priming), and one in which target and distractor swapped their roles (S_rep/T_ch; also considered as full negative priming). We also included a set *change* condition in which the target was repeated, but the distractor was new (S_ch/T_rep; partial positive priming). Lastly, we included a condition that is also normally included in positive and negative priming experiments, but which was not used for comparison with the memory task: a condition in which the set was changed, but the former target became a distractor (partial negative priming). The order of conditions was randomized for each block, and all participants received all conditions (within-subjects design). In total, each condition was tested in five practice and 100 experimental trials. Each of the five drawings appeared as a target and distractor in approximately 20% of all trials.

**Table 1:** Intertrial conditions of the visual perception task

Condition	Target *i* was…	Distractor *i* was…	Example: (format: Tar/Dis trial *i*−1 – Tar/Dis trial *i*)
S_rep/T_rep	Target *i*−1	Distractor *i*−1	Shell/bucket–shell/bucket
S_rep/T_ch	Distractor *i*−1	Target *i*−1	Shell/bucket–bucket/shell
S_ch/T_rep	Target *i*−1	New	Shell/bucket–shell/dummy
Control	New	New	Shell/bucket–dummy/diamond

### Selective long-term memory task

#### Stimulus material

In the selective LTM retrieval task, the same five line-drawings were used as in the selective perception task (cf. panels A vs. B in Fig. 2). However, here we used three different colorings of each drawing (RGB red, blue, and yellow), yielding 15 different drawing × color combinations. A different four-letter non-word name in capital letters, taken from the ARC Nonword Database (Rastle, Harrington, & Coltheart, 2002), was assigned to each colored drawing, and all names for differently colored versions of the same object began with the same two letters (see Fig. 2b). This served to facilitate the formation of memory networks based on the individual objects. The last two letters of any name were a unique combination of consonants only used in that name to facilitate the discrimination of names within one object category.

#### Procedure

**Fig. 3:**
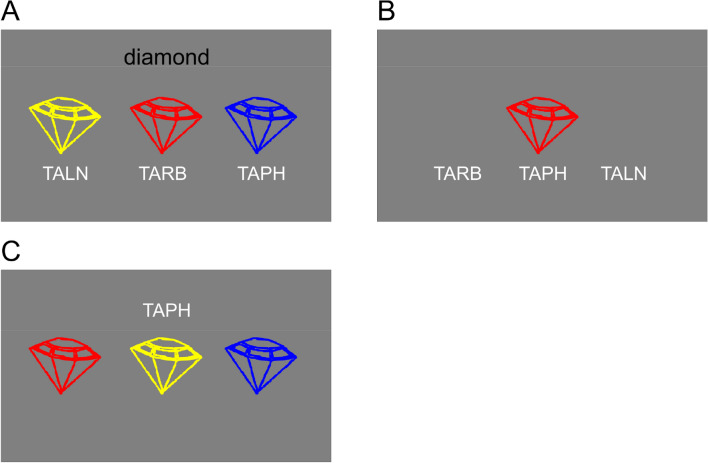
Exemplary trial displays for the three types of additional learning procedures for the selective LTM task. Panel A shows an example display for the initial self-paced initial memorization procedure. Panel B depicts an example display for the multiple-choice quiz that was used to test the learned stimuli, and at the same time represented a second learning procedure. The correct name for the depicted stimulus had to be selected via button press, and feedback for the correct and incorrect choices was provided. Panel C illustrates the multiple-choice quiz that occurred intermittently during the retrieval task. The name of a stimulus was provided as a cue and the correctly corresponding stimulus had to be selected

The task consisted of two phases, a learning phase in which the 15 non-word names and their associated drawings had to be memorized (Fig. 3), and a retrieval phase (Fig. 1b) in which these names had to be either recognized (standard trials) or sometimes unexpectedly recalled (catch trials). At the beginning of the learning phase, all non-word names were presented along with their associated drawings once. To this end, each set of three differently colored drawings of an object was presented (with the objects arranged horizontally) along with the name of the set (e.g., “diamond”) and the objects’ names to strengthen the association between the verbal and visual representations. Participants were instructed to memorize all names together with the associated drawings. The sequence of the five object sets was shuffled for each participant, and learning was self-paced. After participants had memorized all name-drawing pairs (Fig. 3a), they were tested in a multiple-choice quiz (Fig. 3b).

On each quiz trial, one of the 15 objects was presented centrally, while the three different names associated with the respective object set were shown below (see Fig. 3b). Participants had to select the correct name of the colored drawing by pressing either the left mouse button (name on the left), the mouse wheel (name at the center), or the right mouse button (name on the right). The allocation of names to locations was randomized for each trial. Participants were asked to take their time to respond as accurately as possible. The font color of the selected name changed to RGB green after a correct response and to RGB red after an error. Participants were instructed to use this feedback to further improve their memory and to press the space bar to continue with the next trial. The multiple-choice quiz was divided into blocks of 30 trials, with each line drawing being presented twice per block. Within blocks, the sequence of drawings was randomized. After each block, the sum of correct responses was displayed until participants pressed space to start the next block. The multiple-choice quiz was terminated when participants achieved a total of two error-free blocks, which did not have to occur in direct succession. On average, the duration of the complete learning phase (initial memorization and quiz) was 35.43 min (*SD* = 20.05 min, ranging from 5 to 85 min).

After completion of the learning task, the retrieval phase started (Fig. 1b). Each trial started with a centrally presented black fixation cross. After 2000 ms, the name of a set of objects was presented in white on a grey background at the center of the screen and participants were instructed to press the space bar as soon as they had recalled the three associated objects and their names. A verbal color cue (i.e., “red”, “blue”, or “yellow”) was then presented centrally in white for 500 ms to indicate the retrieval target and participants had to decide as quickly and accurately as possible whether the name presented immediately after belonged to the object specified by the color cue or not. Note that verbal cues were chosen instead of the original drawings to avoid perceptual repetition priming and therefore to ensure that the retrieval target had to be actually accessed within the memory network to solve the task successfully. Due to the short presentation time of the color cue, this access was hypothesized to take place primarily during the presentation of the following display.

In 90% of all trials, either the target's or a retrieval competitor’s name was presented after the color cue. The probability of the name being correct was 50%. Participants were instructed to respond as fast and accurately as possible via the right or left mouse button, respectively, to indicate whether the name is correct or incorrect. In case no response was recorded within 1500 ms after stimulus onset, the next trial started automatically. This occurred in *M* = 4.49% (range 0.27–23.26% across participants, practice trials excluded).

The remaining 10% of all trials were “catch” trials. Catch trials were used to ensure that participants truly tried to recall the names of all differently colored objects in response to the presentation of the object-indicating cue. These trials only differed from the other retrieval trials regarding the last display (see Fig. 1b). In these trials, a question mark appeared instead of a non-word name. Participants were instructed to say the retrieval target’s name aloud as quickly and accurately as possible. Immediately after, they had to press the space bar. If no response occurred 2500 ms after question mark onset, the next trial started automatically. Participants reached this response timeout in *M* = 19.90% of all catch trials, ranging from 0 to 75.61% across participants. The short response deadline was implemented to prevent participants from adopting a task strategy in which memory search for the retrieval target’s name would be initiated only during the presentation of a question mark. All in all, these values show that the participants overall managed to quickly assess the relevant memory representations in most of the trials, which was also further confirmed by post-experimental debriefings. However, the fact that some participants had trouble with the response deadline also underlines the importance of refreshing the motivation to always retrieve all names and visual LTM representations when indicated.

The retrieval phase consisted of 21 blocks à 21 trials. Between blocks, participants completed additional multiple-choice quiz sessions (five trials per session, 100 in total; see Fig. 3c) to increase the likelihood of co-activating the visual representations of the memorized objects together with their names. Such co-activation was desired to strengthen the representation of the competing items, thus increasing the comparability to the selective perception task, in which the distractor is always presented together with the target. In contrast to the multiple-choice quiz during the learning phase (Fig. 3b), the name of a stimulus was presented, and the corresponding image should be selected (Fig. 3c). Feedback was only provided after the practice block, but not during the retrieval phase proper.

The first block of the retrieval phase and the following first multiple-choice quiz served as practice trials and were not included in the data analysis. In these trials, feedback was provided by displaying the word “Error” centrally for 1000 ms in red after every incorrect response. The retrieval phase took approximately 80 min.

#### Design

To examine trial-by-trial effects of attentional selection during LTM retrieval, we systematically varied whether the activated associative memory network and the relevant target color were repeated or changed from trial *i*−1 to *i*. On each block’s first trial, both the set and target feature were chosen randomly from the pool of available options. The remaining 20 trials per block were assigned to the following four conditions, yielding five trials per condition: In the S_rep/T_rep condition, both the set of objects and the target feature of trial *i*−1 were repeated in trial *i*. In the S_rep/T_ch condition, the set was repeated, while the target feature changed. In this condition, the retrieval target in trial *i* had been a competitor in trial *i*−1, while the target of trial *i*−1 became a competitor in trial *i*. In the S_ch/T_rep condition, the relevant network changed*,* but the feature was repeated. In this condition, the identities of retrieval target and competitors changed. However, the repetition of the target’s feature possibly facilitated the target activation due to feature priming. Lastly, the S_ch/T_ch condition served as a control condition in which both the network and the feature changed. The order of conditions was randomized within blocks. As in the perception task, each condition was tested in a total of five practice trials and 100 experimental trials. Each network was accessed in approximately every fifth trial (20% of trials), while each target feature was used in approximately every third trial.

### Comparison of tasks

To directly compare the selective perception and the selective LTM retrieval task, the experimental conditions were matched as closely as possible, as listed in Table 2. Matching was based on two factors: First, whether the set of potentially relevant internal/external representations changed or was repeated from trial *i*-1 to *i*, and second, whether the target that had to be selectively attended was changed or repeated. In the selective perception task, a set of potentially relevant representations were the two drawings presented simultaneously in the discrimination display. The target was the drawing in which the gap had to be found. In the selective retrieval task, a set of potentially relevant representations were the three differently colored drawings of an object that were internally represented after the presentation of the object-indicating cue. The target was the name of the drawing that was indicated by the color-indicating cue.

**Table 2:** Matching between conditions of the selective perception versus selective LTM retrieval task (see main text for details)

Target	Set of coactive representations	
	Repeated	Changed
Repeated	S_rep/T_rep (full positive priming)	S_ch/T_rep (partial positive priming)
Changed	S_rep/T_ch (full negative priming)	S_ch/T_ch (control)

### Statistical analysis

All analyses were done using SPSS version 25 (IBM, Armonk, New York, USA). Pairwise comparisons were carried out using dependent *t* tests. Cohen's *d* will be reported as a measure of effect size, using Neath's online tool for within-subjects designs (https://memory.psych.mun.ca/models/stats/effect_size.shtml, access date: 2019-05-15) that computes Cohen's *d* as $=\frac{\left|{m}_{1}-{m}_{2}\right|}{\sqrt{{(s}_{1}^{2} + {s}_{2}^{2} - \left(2r{s}_{1}{s}_{2}\right)}}$. Multivariate analyses were conducted using repeated-measures analyses of variance. Effect sizes for those analyses will be reported as partial eta squared (*η*_*p*_^2^). For all analyses, practice trials and trials during which a timeout was reached were excluded. In all RT analyses, only correct responses were included.

## Results

**Fig. 4:**
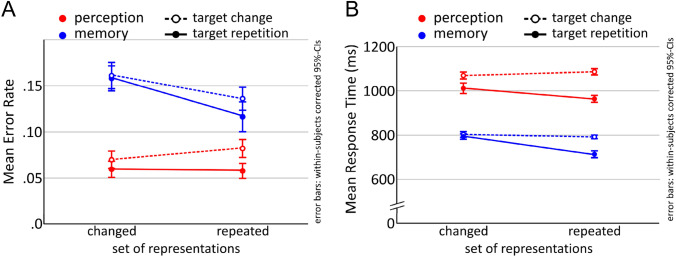
Task performance in the visual attention and memory retrieval task. Error rate is shown in panel A and mean RTs in panel B. Attentional switching in memory and perception are associated with similar performance patterns, but target repetition had a larger impact on selection in perception while set repetition was more for the selection in memory

To keep the results comparable, we performed 2 × 2 ANOVAs for the perception and memory tasks, including factors Set (change, repetition) and Target (change, repetition). An overview of the results of experiment 1 for both perception and memory task performance is depicted in Fig. 4.

### Selective perception task: classical positive and negative priming effects

#### Error rate

Consistent with the literature regarding positive and negative priming, mean error rate across conditions showed the following pattern (see Table 3): S_rep/T_ch (full negative priming) > S_ch/T_ch (control) > S_ch/T_rep (partial positive priming) = S_rep/T_rep (full positive priming). Please note that this only refers to the descriptive, not inference statistics. The statistical analysis of error rates revealed no significant main effect of Set [*F*(1,22) = 1.10, *p* = 0.307, *η*_*p*_^2^ = 0.047], but a highly significant main effect of Target [F(1,22) = 12.11, p = 0.002, *η*_*p*_^2^ = 0.355] with lower error rates for target repetitions. The interaction between both factor was not significant [*F*(1,22) = 1.83, *p* = 0.190, *η*_*p*_^2^ = 0.077].

**Table 3:** Error rates and response times for the selective perception task of experiment 1

Condition	Error rate		Response time	
	Mean	SD	Mean (ms)	SD (ms)
S_rep/T_rep	.06	.01	963	209
S_rep/T_ch	.08	.01	1087	227
S_ch/T_rep	.06	.01	1011	238
S_ch/T_ch (control)	.07	.01	1070	235

#### Response time

Mean RT (in response to the display in which the gap in the target drawing had to be found), showed the following pattern (see Table 3): S_rep/T_ch > S_ch/T_ch (control) > S_ch/T_rep > S_rep/T_rep. In other words, target repetition reduced RTs, but as soon as a previous distracter became a target, it prolonged them. The RT analysis showed only a marginally significant effect of Set [*F*(1,22) = 3.58, *p* = 0.072, *η*_*p*_^2^ = 0.140], but a highly significant effect of Target [*F*(1,22) = 62.45, *p* < 0.001, *η*_*p*_^2^ = 0.739] a well as a highly significant interaction [*F*(1,22) = 13.89, *p* = 0.001, *η*_*p*_^2^ = 0.387]. This is due to the beneficial effect of target repetition being larger within than across sets, i.e., conditions in which the set changed (S_ch/T_ch and S_ch/T_rep) differed less from each other than those in which the set was repeated (S_rep/T_ch and S_rep/T_rep). Pair-wise comparisons against the control condition revealed a significant difference for S_ch/T_ch and S_rep/T_rep [*t*(22) = 8.60, *p* < 0.001, *d* = 1.802], as well as S_ch/T_rep and control [*t*(22) = 3.31, *p* = 0.003, *d* = 0.697], but not for S_ch/T_ch and S_rep/T_ch [*t*(22) = 1.74, *p* = 0.095].

### Selective long-term memory task

To evaluate whether the associations between drawings and non-words had been successfully learned, we determined the mean accuracy in the multiple-choice quiz following the initial memorization phase. This was 0.95 (SD = 0.05), indicating an overall sufficient learning of the items and their names. Mean accuracy for the main task was 0.86 (SD = 0.09).

#### Error rate

The conditions of the memory task revealed the following pattern (see Table 4): S_ch/T_ch (control) = S_ch/T_rep > S_rep/T_ch > S_rep/T_rep. As expected, repeating both the set of potentially relevant memory representations and the target representation resulted in the lowest error rate, while the highest error rate was found for the control condition in which both changed. A 2 × 2 repeated-measures ANOVA with factors Set (change, repetition) and Target (change, repetition) revealed a significant main effect of Set [*F*(1,22) = 10.54, *p* = 0.004, *η*_*p*_^2^ = 0.324] and a marginal main effect of Target [*F*(1,22) = 3.53, *p* = 0.074, *η*_*p*_^2^ = 0.138]. The interaction was not significant [*F*(1,22) = 1.55, *p* = 0.227, *η*_*p*_^2^ = 0.066].

**Table 4:** Error rates and response times for the selective memory task for experiment 1

Condition	Error rate		Response time	
	Mean	SD	Mean (ms)	SD (ms)
S_rep/T_rep	.12	.02	714	90
S_rep/T_ch	.14	.02	792	99
S_ch/T_rep	.16	.02	793	111
S_ch/T_ch (control)	.16	.02	803	102

#### Response time

Regarding mean RTs (see Table 4), the pattern was S_ch/T_ch > S_ch/T_rep > S_rep/T_ch > S_rep/T_rep. The shortest mean RT was found for the condition in which both the set of associations and the target was repeated, while the control condition in which both changed was associated with the highest mean RT. A 2 × 2 repeated-measures ANOVA like the one for memory accuracy revealed significant main effects of Set [*F*(1,22) = 27.91, *p* < 0.001, *η*_*p*_^2^ = 0.559] and Target [*F*(1,22) = 67.52, *p* < 0.001, *η*_*p*_^2^ = 0.754], as well as a significant interaction [*F*(1,22) = 29.21, *p* < 0.001, *η*_*p*_^2^ = 0.572]. Paired comparisons of all conditions against the control condition S_ch_/T_ch revealed only a statistically significant difference for S_rep/T_rep from S_ch_/T_ch [*t*(22) = 7.46, *p* < 0.001, *d* = 1.558]. There was neither a statistically significant difference between S_ch/T_ch and S_ch/T_rep [*t*(22) = 1.15, *p* = 0.264], nor between S_ch_/T_ch and S_rep/T_ch [*t*(22) = 1.27, *p* = 0.216].

## Discussion

In the first experiment, our focus was on matching the experimental stimuli and selection demands as closely as possible, while preserving the inherent structure of the classical positive/negative priming and selective long-term memory retrieval experimental designs. One problematic aspect we tried to address was that while representations of the stimuli are processed online for perception, they first have to be activated in memory to perform any additional (selection) processes on them. Hence, to ensure that only the selection was carried out during the critical display, retrieval (or perception) of all potentially relevant stimuli (targets and distractors) was carried out at the beginning of each trial, prior to the cue indicating the target.

The result patterns found for the perceptual task, where the repetition of the target was the driving factor, are in sharp contrast to the memory task where we found a highly significant main effect of set repetition, but only a marginal effect of target repetition. In the memory task, error rates were lower when the set of potentially relevant representations was repeated, even when the former target became a retrieval competitor and vice versa, however, this was not the case for the perceptual task. In other words, for memory, it is the repetition of the set of representations while for perception, it is the repetition of the target that proved to be more beneficial. To conclude, switching the target within a pre-activated set is detrimental only for perceptual selection, but not for selection in memory, where the shifting of the set is more detrimental.

However, processing differences may also have occurred due to the differing number (one versus two) and nature of distractors (different uncolored objects vs. different colors of the same object). We had chosen only one distractor for the perception task, because it is the standard for positive and negative priming tasks, such as the one by Tipper and Driver (1988), and because more than one distractor may have made the display too crowded. We also had refrained from using different colors in the perception task, because it is well-established that color is a more prominent feature than form for selective attention in visual perception, even enabling parallel search (Alexander, Nahvi, & Zelinsky, 2019; Kopp, Tabeling, Moschner, & Wessel, 2007; Turatto & Galfano, 2000). Hence, selecting according to color seemed too easy and therefore not comparable to the memory task. Nonetheless, to rule out that these differences between tasks could have been the leading factors for the diverging result patterns between perception and memory, we conducted a second experiment.

## Experiment 2

**Fig. 5:**
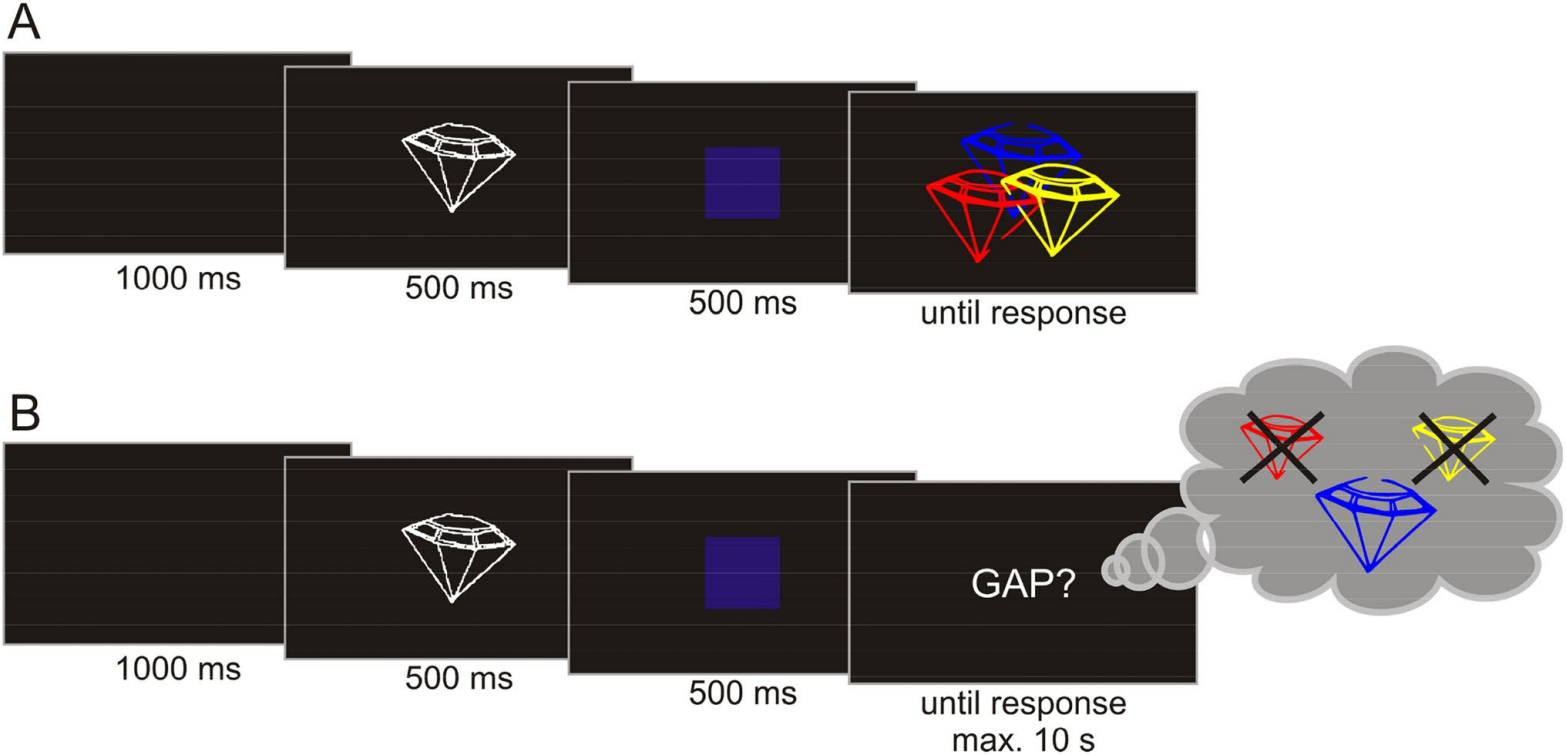
Exemplary trials for selection in perception (panel A) and memory (panel B). Cueing was done perceptually for both tasks (first category, then target color), while providing too little time for intentional preactivation of memory representations. In both tasks, the spatial location of a gap in the presented or remembered line drawing for the indicated target stimulus had to be provided via button press

In an attempt to parallelize the selective perception and memory tasks even further, we used exactly the same stimuli for targets and distractors, as well as the exact same task (see Fig. 5). The task in both domains now was to indicate the location of a gap in the indicated target stimulus, which either had to be focused on during perception or selectively retrieved from memory (Fig. 5). There were two distractors in both tasks and all distractors were of the same object as the target, but in different colors.

## Methods

### Participants

Twenty-three participants (15 female, 8 male) between the ages of 20 and 44 (Median: 22, SD:5 years) with normal or corrected-to normal vision gave their informed consent to take part in the experiment and were paid 8 euros per hour or were rewarded with credit points for their participation. Normal colour perception was assessed with Ishihara plates for colour perception (Ishihara, 1972). Two participants were not included in the analysis due to less than 60% correct responses in the perception task, suggesting that instructions had been misunderstood. The final sample consisted of 14 females and 7 males between 20 and 44 years (Median: 22, SD: 5.2 years, 3 left-handed, 18 right-handed).

### Selective perception task

#### Stimulus material

**Fig. 6:**
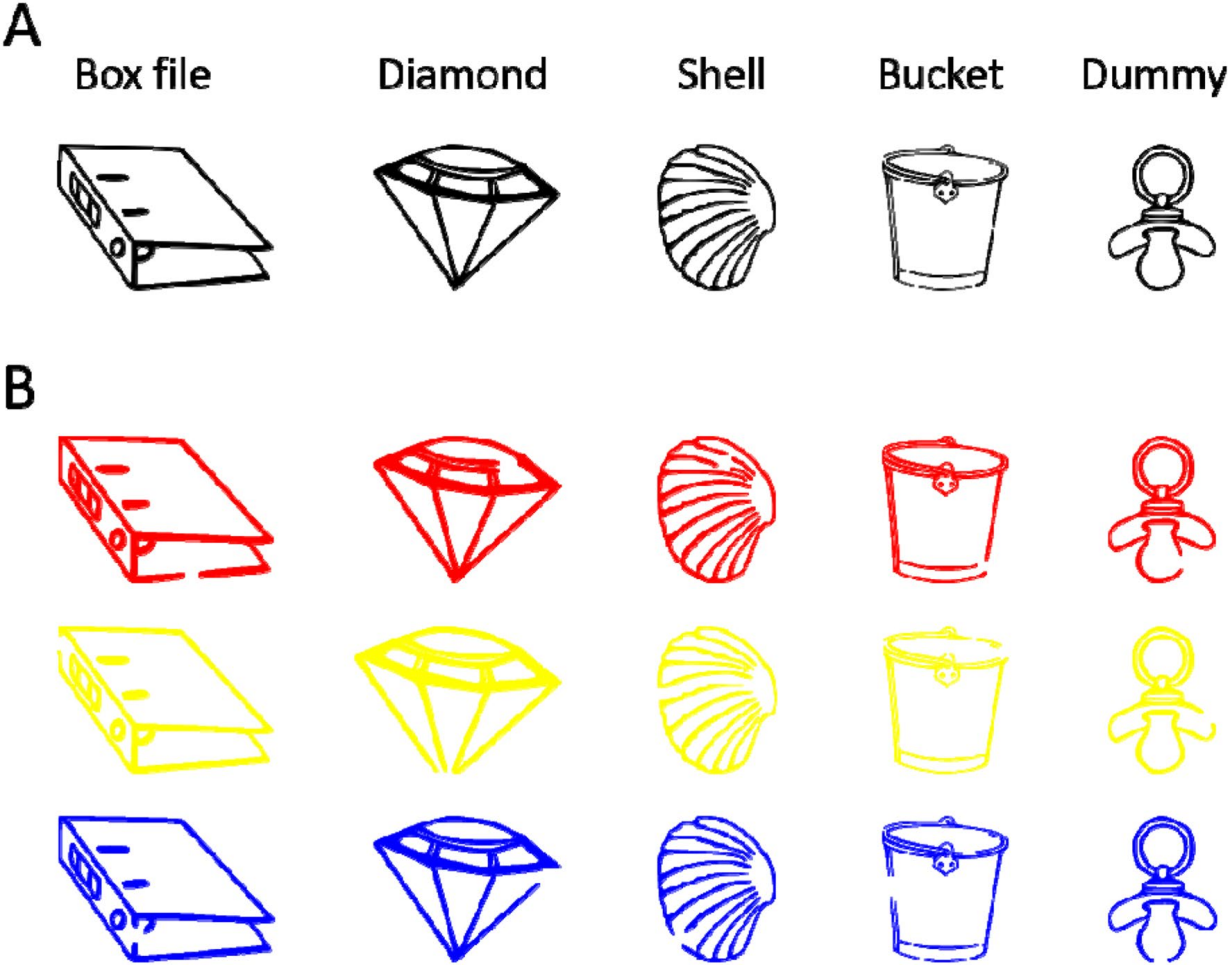
Stimuli used in experiment 2 for both the perception and memory task. Panel A depicts all five categories of line drawings, panel B an example set of stimuli used in the search display. Targets and distractors were always chosen from the same category, that is, all three colors were presented

The same five objects in the three different colors as in Exp. 1 were used, with a small gap with a height of 0.13 cm inserted at one out of eight possible positions (Fig. 6). All stimuli were presented on a black background.

#### Procedure

Experiment 2 was programmed and presented using Presentation 21.1 (Neurobehavioral Systems). The software was also used to record all responses. Otherwise, the testing environment was identical to experiment 1. Each trial started with the presentation of a fixation cross in the middle of a screen for 1000 ms, followed by the category cue (i.e., a white object without gap) for 500 ms. Immediately after, the target color was cued by a small colored square presented for 500 ms, followed by the presentation of the target object. The participant’s task was to indicate the target’s gap location as quickly as possible by pressing one out of eight buttons from the keyboard’s number pad that were consistent with the gap position (e.g., bottom left = 1, top middle = 8, etc.) while ignoring the superimposed differently colored objects (distractors). The gap locations were randomly selected and were different for all three objects (the target and the two distractors). The objects’ centroids were randomly located on an imaginary circle with a radius of 70 pixels to prevent an anticipatory allocation of spatial attention to a specific location.

In total, 400 trials were presented in blocks of 50 trials, preceded by a practice block, during which feedback was given as either the correct target object presented without distractors (after incorrect responses) or a smiley face giving a thumbs up (after correct responses).

#### Design

A within-subjects 2 × 2 factorial design with factors Set (repetition, change) and Target (repetition, change), comprised the following conditions:

1. Set and target change (S_ch/T_ch), with both the object category and the color of the target changing from trial *i*−1 to trial *i*. This condition was regarded as the *control* condition.
2. Set change and target repetition (S_ch/T_rep), with the object category changing while the target color remained unchanged. The condition was regarded as partial positive priming.
3. Set repetition and target change (S_rep/T_ch), with the object category remaining unchanged, while the target color changed, i.e., a former distractor became the target and the former target a distractor. This was regarded as full negative priming.

Set repetition and target repetition (S_rep/T_rep), with both the object category and color of the target remaining unchanged. This condition was regarded as full positive priming.

### Selective long-term memory task

#### Stimuli

The same stimuli were used as for the perception task (Fig. 6). Participants were instructed to memorize 15 stimuli,^3^ consisting of all object-color combinations with gaps at specific locations randomly chosen for each participant with the constraint of different gap locations at least within each object category, and no gap location appearing more than twice.

#### Procedure

The trial sequence of the selective retrieval task was identical to the perception task except for the last display. Here, instead of three objects the question “*GAP?*” appeared in the center of the screen, upon which the participant had to selectively retrieve the respective gap location and press the corresponding button on the number pad as quickly as possible. A timeout of 10 s was introduced to account for cases of retrieval failure.

**Fig. 7:**
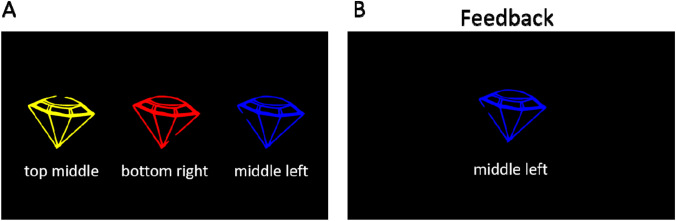
Exemplary trials of the learning phase of experiment 2. Panel A shows an exemplary display of the initial self-paced memorization procedure. Panel B shows the additional feedback display used of the retrieval-practice session which was provided in addition to the trial structure depicted in Fig. 5b, but left out of the experiment proper

The retrieval task was preceded by two learning phases to memorize the objects and their gap locations. In the first phase, each of the five sets was presented with the gap locations written out below the objects (see Fig. 7a) and participants could repeatedly (at least three times) contemplate the sets at their own pace to memorize them until they said that they felt confident to go into a memory test for the objects.

In the second phase, a modified version of the actual selective retrieval task was utilized as a retrieval-practice session. After the presentation of the category cue and color cue for the target, the participant was asked to recall the gap’s position of the cued object by pressing the corresponding button on the number pad. Participants were provided with feedback by means of a smiley face giving a thumbs up following correct answers or the target stimulus with its gap location in written format below the drawing following incorrect answers (see Fig. 7b). To increase the associations between the objects within each category, all stimuli of each category were tested in direct succession. The order of categories was shuffled. The exercise was completed when at least three repetitions of the 15 stimuli had been completed (45 trials), with at least two consecutive blocks with less than two errors.

#### Design

The design was identical to the perception task.

### Statistical analysis

Statistical analysis was the same as in Exp. 1. For the analysis of RTs, trials with incorrect answers and in which the response timeout for the memory task was reached (after 10 s) were excluded. A 2 × 2 repeated-measures ANOVAs and dependent-measures *t* tests were applied for both the RTs and error rates.

## Results

### Selective perception task

#### Error rate

**Fig. 8:**
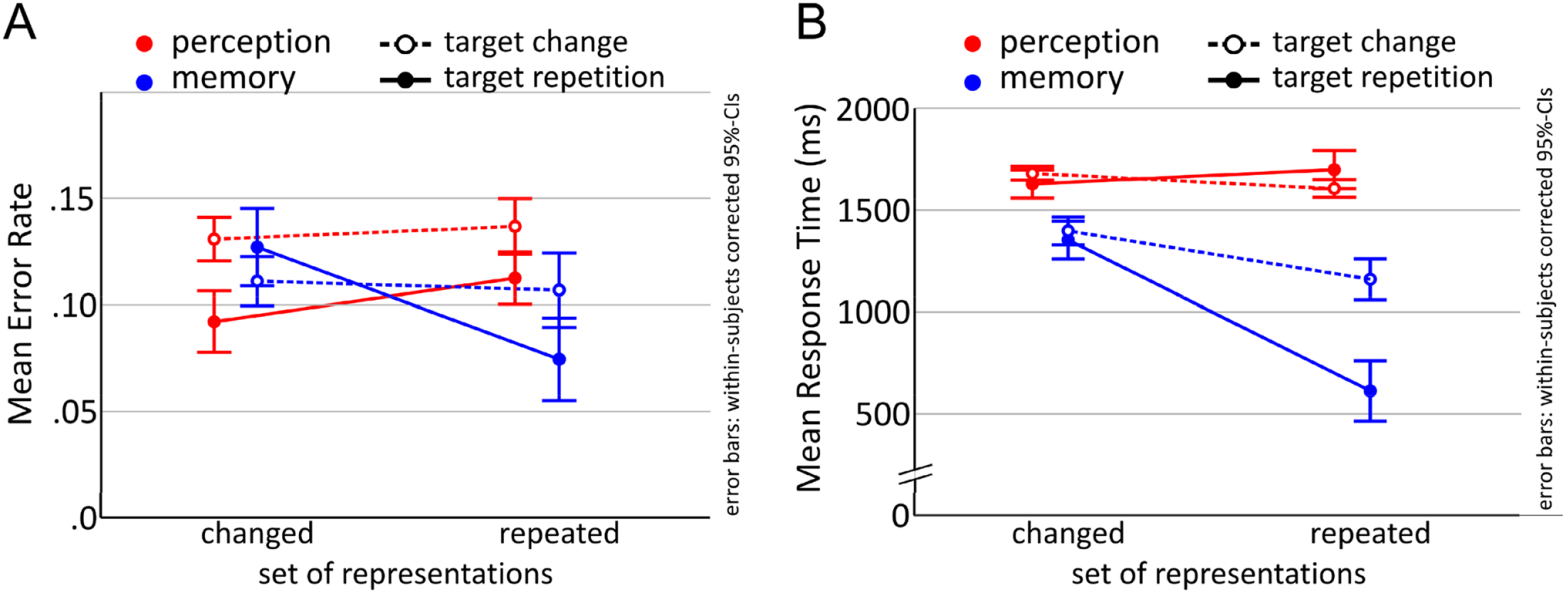
Behavioral performance for the selective perception and selective retrieval tasks. Both error rate (**a**) and mean RTs (**b**) show clearly different patterns, again mainly attributable to a main benefit from target repetition (T_rep) for perception, while behavioral effects for memory were strongly dependent on set repetition

Consistent with the literature regarding positive and negative priming, mean error rate across conditions descriptively showed the pattern S_rep/T_ch (full negative priming) > S_ch/T_ch (control) > S_rep/T_rep (full positive priming) > S_ch/T_rep (partial positive priming) (Table 5; Fig. 8a). A 2 × 2 repeated-measures ANOVA with factors Set (repetition, change) and Target (repetition, change) revealed significant main effects of both Set [*F*(1,20) = 6.43, *p* = 0.020, *η*_*p*_^2^ = 0.243], with lower error rates for set change compared with set repetition, and Target [*F*(1,20) = 16.69, *p* = 0.001, *η*_*p*_^2^ = 0.455], with lower error rates for target repetition. The interaction was not significant [*F*(1,20) = 0.88, *p* = 0.360, *η*_*p*_^2^ = 0.042].

**Table 5:** Error rates and response times for the selective perception task for experiment 2

Condition	Error rate		Response time	
	Mean	SD	Mean (ms)	SD (ms)
S_rep/T_rep	.11	.07	1697	683
S_rep/T_ch	.14	.06	1604	466
S_ch/T_rep	.09	.07	1627	411
S_ch/T_ch (control)	.13	.08	1678	506

#### Response time

Mean RTs were compared using the same 2 × 2 ANOVA. In accordance with the small and inconsistent descriptive differences, as presented in Table 5, the analysis revealed no significant effects (Fig. 8b).

### Selective long-term memory task

#### Error rate

Mean accuracy for the selective retrieval task was 0.90 (0.08), thus overall recall success was very high. Error rates for the memory data were analyzed using the same 2 × 2 repeated-measures ANOVA as for the perception data. The analysis revealed a significant main effect of Set [*F*(1,20) = 9.01, *p* = 0.007, *η*_*p*_^2^ = 0.311], indicating that set repetition was associated with generally lower error rates than set change, as well as a significant interaction [*F*(1,20) = 5.51, *p* = 0.029, *η*_*p*_^2^ = 0.216], suggesting that target repetition only had an effect in set repetition (see Fig. 8a). Post hoc comparisons of all conditions against the control condition support this visual impression, as there was a highly significant difference between S_ch/T_ch and the full positive priming condition (S_rep/T_rep) [*t*(20) = 2.83, *p* = 0.010, Cohen’s *d* = 0.617], but no significant difference between S_ch/T_ch and S_ch/T_rep [*t*(20) = 1.03, *p* = 0.314, Cohen’s *d* = 0.225], nor between S_ch/T_ch and S_rep/T_ch [*t*(20) = 0.52, *p* = 0.608, Cohen’s *d* = 0.113].

#### Response time

Mean RTs showed the following pattern (see Table 6; Fig. 8b): S_ch/T_ch (control) ≥ S_ch/T_rep (partial positive priming) > S_rep/T_ch (full negative priming) > S_rep/T_rep (full positive priming). A 2 × 2 repeated-measures ANOVA revealed significant main effects of Set [*F*(1,20) = 50.51, *p* < 0.001, *η*_*p*_^2^ = 0.716] and Target [*F*(1,20) = 32.86, *p* < 0.001, *η*_*p*_^2^ = 0.622], as well a significant interaction [*F*(1,20) = 22.11, *p* < 0.001, *η*_*p*_^2^ = 0.525]. Corresponding to the error rates, set repetition was associated with generally lower RTs than set change, but target repetition only had an effect in set repetition, which is, in contrast to the error rates, strongly supported by the post hoc tests. When compared to the control condition S_ch/T_ch, S_ch/T_ch did not differ from S_ch/T_rep [*t*(20) = 1.16, *p* = 0.260, Cohen’s *d* = 0.192], but there was a highly significant difference between S_ch/T_ch and the full positive priming condition (S_rep/T_rep) [*t*(20) = 8.25, *p* < 0.001, Cohen’s *d* = 1.775], as well as for S_ch/T_ch and S_rep/T_ch [*t*(20) = 3.71, *p* = 0.001, Cohen’s *d* = 0.810].

**Table 6:** Error rates and response times for the selective memory task for experiment 2

Condition	Error rate		Response time	
	Mean	SD	Mean (ms)	SD (ms)
S_rep/T_rep	.07	.08	610	232
S_rep/T_ch	.11	.09	1155	579
S_ch/T_rep	.13	.09	1347	582
S_ch/T_ch (control)	.11	.10	1392	559

## Discussion

The second experiment focused on fully parallelizing the exact number and appearance of the stimuli for the perception and memory tasks, as well as the response, while systematically repeating or changing the underlying set of potentially response-relevant internal/external representations. We generally replicated the result pattern from Exp. 1 for selection in memory, which again showed that target repetition only had an effect when it occurred in conjunction with set repetition. In other words, there was no positive priming of a single feature (color) alone, only of the integrated object representation. Set repetitions decreased RTs and reduced error rates, but to a much higher degree when stimulus set and target color were repeated. Thus, selection in memory benefits from repetitions of the previously activated set of memory representations, and even more so from repetitions of set and retrieval target.

This finding is in contrast to the perception data. Here, targetcolor and stimulus set repetitions affected error rates without interaction. Surprisingly, repetitions of target color had a more beneficial effect of reduced error rates when the stimulus set changed (i.e., different line drawings; S_ch/T_rep), then when the set was repeated (i.e., same line drawings; S_rep/T_rep). This was unexpected, because the S_rep/T_rep condition can be considered full positive priming, because distractors and targets both were repeated and kept their roles, whereas S_ch/T_rep meant that only the target color was repeated, whereas the stimuli themselves were changed (partial positive priming). However, this finding can be reconciled with the literature on positive priming, when considering that the actual response to the target, i.e., the specific press on one of the 8 response buttons, was only repeated in 1/8 of the trials,^4^ because the location of the gap that needed to be found was always chosen randomly from 8 different options. Hence, even when all stimuli including the identities of the targets and distractors were repeated, the response still changed in most cases. According to the stimulus–response retrieval account of priming (Frings, Schneider, & Fox, 2015), which proposes that advantages of target repetitions (and distractor) are mainly due to repetitions of stimulus–response associations, our full positive priming condition S_rep/T_rep should not lead to pronounced priming at all, because the exact stimulus–response mapping is repeated in only 1/8 of the trials. In fact, it could rather lead to substantial retrieval interference with the previous stimulus–response mapping of the same stimulus. Now in the S_ch/T_rep condition, all stimuli are changed, not cuing any previous stimulus–response associations, so any detrimental effect due to interfering stimulus–response mappings should be absent, whereas, at the same time, there might still be benefit from focusing on the same color. However, since response repetitions have not been systematically varied here, possible effects of differences in the number of response repetitions have to be investigated in a follow-up study.

## General discussion

In the current study, we compared attentional selection in memory versus perception within participants, using the same stimulus material and a closely corresponding trial structure in both domains. Specifically, in two experiments, the set of stimuli on which attentional selection had to be performed was either presented visually or had to be activated in memory. Out of this set, the target was indicated, that is, selective attention had to be applied, and a decision had to be made. We were interested in the effects of applying cognitive-control processes (selective attention towards the target and possibly the inhibition of distractors) that can be measured on a trial-by-trial basis.

Across both experiments, a relatively coherent behavioral pattern emerged for perception and memory. For selection in perception, we found a consistent benefit for target repetition (for error rates in both experiments, for RTs in experiment 1), not just for a repetition of the whole target object, but also for a repetition of merely the target’s color, even with different objects. Importantly, this benefit cannot be attributed to response repetition, because the likelihood of a response repetition for any condition, even when target and distractors were repeated, was 50% in Exp. 1 and only 12.5% in Exp. 2. Hence, the benefit is most likely due to a benefit from refocusing on the same object and/or color. Although we did find a small disadvantage for the classical negative priming condition in terms of slightly increased error rates for set repetition when target and distractor swapped their roles (S_rep/T_ch) in Exp. 1, there was no indication of negative priming in Exp. 2. Hence, we found no clear evidence for distractor inhibition (Mayr & Buchner, 2007; Tipper, 2001).

In contrast, for selection in memory, set repetition seemed to be the driving force behind all beneficial effects, while there was no condition which showed weaker performance as compared with the control condition. In both experiments, we found behavioral benefits of repeating the set of relevant memory representations. In Exp. 1, error rates for both set repetitions were reduced, that is, even the condition S_rep/T_ch, in which a former distractor became the target (and vice versa), showed a reduction in error rates compared to the control condition, although not as much as the condition in which set and target were repeated (S_rep/T_rep). This finding is a replication of our previous study, in which we also varied fast attentional switching from trial to trial in a selective LTM retrieval task (Kizilirmak et al., 2014) and suggests that the benefit of having already retrieved and activated the LTM contents outweighed any potential detrimental effects of selectively focusing on a previous retrieval competitor. This benefit further seems to be independent from the instruction to retrieve only one or all associations with a cue in trial *i*−1, which we could show in another study in which we also manipulated the number of retrieval targets from trial *i*−1 to *i* (Kizilirmak, Rösler, Bien, & Khader 2015). Such a finding is highly surprising in terms of an inhibitory account of negative priming (for a discussion, see Frings et al., 2015; Schrobsdorff, Ihrke, Behrendt, Herrmann, & Hasselhorn, 2012; Tipper, 2001), because one would expect interference to be stronger the more recent the activation of distractors is (or retrieval competitors in this case). In RTs, we again found no detrimental effects of shifting selective attention to a previous distractor. Mean RT for the only condition that significantly differed from the others was the one in which both set and target were repeated (S_rep/T_rep). Because we took care to that the whole set of memory representations was pre-activated intentionally in Exp. 1, this result is probably due to the fact that only this condition had the additional advantage of selecting exactly the same representation again. In line with this interpretation, in Exp. 2, where the set was not pre-activated before the critical selection display, RTs showed a significant reduction for *both* set repetition conditions. Thus, the processing advantages of prior activations of associated representations and repeated selections of one and the same target representation could be dissociated. To summarize, we again only found beneficial effects of repetitions of the set and an additional advantage on top when set and target were repeated.

Coming back to the main question at hand, that is, whether similar control mechanisms are involved in selection in memory and perception, the diverging result patterns suggest that there is an overlap in terms of benefitting from re-attending to a target representation, but whereas in perception, just refocusing on the same target or target color is already associated with a general behavioral advantage, target repetitions are only of note for memory if it is the very same memory representation (not just a feature like color) which needs to be re-attended. In memory, any advantages seem to mainly depend on repetitions of the set of representations, even when it is the previous distractor that becomes the target.

We propose that there are two main factors playing an important role in selection in memory and perception which differently affect selection in the two domains. The main factor seems to be an additional process that comes on top of the selection, but only in memory: The initial activation or retrieval of the memory representations. Whereas stimulus representations in perception are processed *online*, while selection takes place in representations of stimuli that are being currently perceived, in memory, those representations are processed *offline*, and have to be first retrieved and second actively held in working memory (Exp. 1) while selection takes place *or* target and distractors are being activated (intentionally and automatically via spreading activation) during the process of selective retrieval. In the LTM task, attentional resources are already deployed to a substantial degree to search for and reactivate a set of potentially task-relevant LTM representations, and to keep them active in working memory (WM). There is evidence that the same attentional resources are involved in holding WM representations active (i.e., activated LTM contents; internal stimuli) and attending towards external stimuli (Chun, 2011; Kiyonaga, Dowd, & Egner, 2017; Kiyonaga & Egner, 2014). The resource-demanding initial search for the respective LTM representations requires cognitive resources that are not necessary, at least not as much, in the visual perception task, leading to the observed general benefit of repeating sets of representations independent of stimulus–response associations. This leads to a general advantage of repeating the same set of stimulus representations in the memory task, which became only evident in the error rates for Exp. 1, in which we attempted to separate the step of activating the set of potentially relevant representations before the critical selection interval, and also in the RTs in Exp. 2, where this process had to be carried out during the selection interval.

The second factor which is able to explain a main overlap is facilitation for re-focusing on the previously attended target, in the selective attention (in perception) literature referred to as positive priming (e.g., Stadler & Hogan, 1996). The condition in which the stimulus set and target, meaning both target and distractors, are repeated shows a general advantage in error rates as well as in RTs for both domains, memory and perception. However, whereas in memory, only identity priming was observed (same target representation, exp. 1 + 2) in the perception task, even feature priming (of color) in the absence of identity priming (different line drawing, same color) could be observed (perception task, exp. 2). One explanation would be that color is a more dominant feature for the direction of selective attention in perception than in memory, while in memory grouping, features according to semantic meaning (here: objects) are more relevant. The visual attention literature (Lamy & Egeth, 2003; Maunsell & Treue, 2006; Turatto & Galfano, 2000) as well as literature on associative memory networks seems to support this idea (Anderson, 1976; Collins & Loftus, 1975; Ghosh & Gilboa, 2014; Saxe, McClelland, & Ganguli, 2019).

While we took great care to match the stimuli and how to respond to them (i.e., the stimulus material, task, target-distractor space, and even the response options) across selection domains, recent work suggests that whatever is perceived and whichever action is being performed becomes bound into an episodic event file, which might be the basis for subsequent priming (theory of event-coding; Frings et al., 2020; Hommel, Müsseler, Aschersleben, & Prinz, 2001; Singh, Moeller, & Frings 2016). Accordingly, not only the stimuli and responses, but also the specific stimulus–response bindings should be matched across domains to make the selection task as comparable as possible. In the present study, the associated response with a specific stimulus (e.g. the red shell), that is, the set-target combination, was always the same for the memory task, while the response changed in 7/8 of all trials in the perception task. This should be held constant in future studies that compare priming in memory and perception.

## Conclusion

All in all, we propose, based on two experiments, that any attempt of modeling attention to external versus internal stimulus representations in exactly the same way has to cope with issues of comparability that are inherent to the different cognitive processes underlying attentional selection in external versus internal sensory space. As outlined above, we see a substantial difference with respect to the process of activating a set of stimulus representations for attentional selection in the first place. Future studies could try to render this process easier in the memory task or more difficult in the perceptual task, e.g., by presenting stimuli within a certain amount of visual noise, making them harder to perceive and to discriminate, thus creating similar attentional demands for the initial representation as in the memory domain. Another factor is the larger benefit from re-attending the same target color in perception compared with memory. To match selective perception and memory tasks even further, one would need to carefully figure out target features of similar relevance for search in the sensory environment and in memory. An important aspect which needs to be additionally taken into account, and which seems often to be neglected in cognitive research, are response repetitions and changes potentially confounded with the conditions which manipulate the shifting of the focus of internal or external attention.

Keeping those challenges in mind, the present study delineated principal similarities and differences of selection processes in both domains: While positive priming from stimulus repetition was found in both selection domains, we found no consistent effects of negative priming when shifting the focus of attention to a previously to-be-ignored stimulus. However, priming in the perception task was mainly due to repetitions of the target feature (here: color), whereas for the memory task, repetitions of the same set of stimulus representations was most important. We propose that the differences can be attributed to a reduced cognitive effort when the now relevant memory representation had already been pre-activated (even as a distractor) in the previous trial. Additionally, our experiments both underscore the importance of taking stimulus–response associations into account, which may be a hidden factor behind differences between domains.

## Footnotes

1 Under certain conditions, this pattern can deviate, and even retrieval-induced facilitation of the Rp^−^ items can be found (Bäuml & Samenieh, 2010; Chan, 2009), but I will not discuss this here.

2 English was chosen for the stimulus descriptions, because many of the students who could participate in the study were international students of the Ludwig-Maximilians-University of Munich, Germany.

3 Note that we refrained from sticking to the same 15 stimuli (just one gap position per category-color combination) for the perception task, because it became evident after a few piloting sessions, that the gap positions were involuntarily remembered, turning the perception task into another episodic memory test.

4 For each stimulus, there were eight possible gap positions which were drawn at random to keep participants from learning object + color + gap positions, which would have made it another memory task.
